# Redacted text detection using neural image segmentation methods

**DOI:** 10.1007/s10032-025-00513-1

**Published:** 2025-01-30

**Authors:** Ruben van Heusden, Kaj Meijer, Maarten Marx

**Affiliations:** Information Retrieval Lab, University of Amsterdam, Amsterdam, The Netherlands

**Keywords:** Text Redaction, Instance Segmentation, Mask R-CNN, Mask2Former

## Abstract

The redaction of sensitive information in documents is common practice in specific types of organizations. This happens for example in court proceedings or in documents released under the Freedom of Information Act (FOIA). The ability to automatically detect when information has been redacted has several practical applications, such as the gathering of statistics on the amount of redaction present in documents, enabling a critical view on redaction practices. It can also be used to further investigate redactions, and whether or not the used techniques provide sufficient anonymization. The task is particularly challenging because of the large variety of redaction methods and techniques, from software for automatic redaction to manual redactions by pen. Any detection system must be robust to a large variety of inputs, as it will be run on many documents that might not even contain redactions. In this study, we evaluate two neural methods for the task, namely a Mask R-CNN model and a Mask2Former model, and compare them to a rule-based model based on optical character recognition and morphological operations. The best performing, the Mask R-CNN model, has a recall of .94 with a precision of .96 over a challenging data set containing several redaction types. Adding many pages without redaction barely lowers this score (precision drops to .90, recall drops to .92). The Mask2Former model is most robust to inputs without redactions, producing the least false positives of all models.

## Introduction

Text redaction refers to the process of removing sensitive information from texts which are made public. This often concerns personal information like names of witnesses in court cases, financial information from contracts, or information released through Freedom of Information Act (FOIA) requests [1, 4]. Text redaction must satisfy two balancing properties. On the one hand, it must be *safe*, text which has to be removed cannot (or only with very small probability) be recovered again from the document. On the other hand, it must be *conservative*, meaning that all other text, layout information and metadata must be kept intact.

Although this seems trivial, a large number of possible ways of redacting information exist, such as using specialized software, blurring- or mosaicing techniques, or even just a black marker pen [14]. Some redaction guidelines^1^,^2^ specifically recommend printing, manually redacting and scanning a PDF document, which means all data on the document-structure and all embedded metadata is lost.

The *detection* of redactions is either a text- or image segmentation task, in which the redacted pieces of text on a page are detected and indicated by their precise bounding box [4]. It can be used for corpus analysis, estimating the number of redactions and the ratio between redacted and visible text. Apart from using this information for gathering corpus statistics, it can also be used to check the quality of the redactions, and whether the text is truly redacted, or still retrievable. Several papers have been published on the vulnerability of different redaction techniques such as black bars, mosaicing and blurring, and have shown that, in specific cases, the original text can be retrieved using either a rule-based or statistical approach [4, 14, 22] The output of a detection system can also be used applied when making released texts accessible according to the WCAG guidelines^3^. When a redacted document is read out loud by software, the redacted pieces simply become a (sometimes quite long) silence, and the spoken sentence is no longer grammatical. By using the detected bounding boxes, redactions can be given an *alt* tag, which can be used by the text-to-speech software to indicate that a piece of text has been redacted.

Redacted texts predominantly come in the form of PDF documents. If these are “digital born”, we have access to the original text in the correct reading order. But very often, redacted texts come as PDFs consisting of *scanned documents*, and we only have access to the underlying text via Optical Character Recognition (OCR). In the latter case, an image-segmentation approach seems the first choice, whereas in the former both approaches and even their combination can be applied. For the intended applications, it is most important to correctly and precisely identify each separate piece of redacted text.

One of the few available approaches for the detection of redacted text is *Edact-Ray* [4], which approaches the task as a text-segmentation task We are aware of only one text redaction detection system, called *Edact-Ray*[4], which approaches the task as a text-segmentation task. Edact-Ray is a rule-based system operating on the text of a page, together with position information of each character. Its heuristic is that when two *consecutive* words are further apart than the width of a space character, the text in between these words has most likely been redacted. The detection algorithm was manually evaluated by the authors, and had a false positive rate of 4%, with no false negatives being detected. It was reasonable to assume that the same idea could work on text obtained by OCR from scans, but, as we will show in this paper, it does not.

van Heusden et al [13] introduce a method for redacted text detection that combines textual information with morphological operations, and evaluate it on Dutch FOIA documents. The method sequentially removes text from an input image, and uses contour detection to detect redactions. Although the technique is successful in detecting redactions of different types, it is not robust when applied to different input sources, yielding detecting a lot of false positives such as parts of images and logos. Although several enhancements are suggested, these would also significantly reduce the ability of the model to detect true redactions.

In an attempt to overcome the disadvantages of a rule-based approach, this paper evaluates two neural image segmentation methods for detecting redactions. The field of image segmentation has seen a rapid development in recent years, with a multitude of models being released for a variety of tasks. These recent models can be roughly grouped into two types, those based on Convolutional Neural Networks (CNNs), and those based on Transformers [24]. Although the latter generally outperforms the former, convolutional approaches sometimes still outperform Transformer-based models on specific domains, which is why a CNN-based model is also included in this research.

The question we will answer here is: *“How well can neural image segmentation models detect redacted text?"*.

We measure performance by comparing detected redactions to ground truth redactions using the panoptic quality methodology [16]. We also look at the effect of the number of training examples on performance, whether we see a difference in performance different performance for different types of redaction, and how our detectors work on “hard negatives" (pages without any redaction).

Our main contribution is a well performing (recall=.94, precision=.96) detection system based on a pre-trained Mask-R-CNN model, which needs a moderate amount of training data (less than one thousand annotated pages), and which also performs well on documents without any redaction. Besides all code, we also release an extensive set of manually labelled train and test data (1,464 pages, 11,572 redactions), grouped by redaction type (see Table 1). Lastly, we show that the heuristic *Edact-Ray*[4], redaction detector does not generalize to text derived from scanned documents by OCR.

The rest of the paper is structured as follows. In Sect. 2, we briefly discuss related work in document segmentation and recent neural instance segmentation models, as well as recent work for automatic detection of redactions. In Sect. 3, we describe the used dataset and methods in detail, including the annotation process and the specific parameters used for the neural methods. Section 4 contains the evaluation of the two neural models in comparison to the rule-based baseline, and also contains several examples. We conclude with a short discussion of the results and possible directions for future work in Sect. 5.

## Related work

### Detection of redacted text

Although previous work on the automatic detection of redacted text is rather limited, several approaches have been proposed in the literature, which use either textual information, or a combination of both textual- and visual information to automatically detect redactions. Bland et al [4] developed the Edact-Ray Tool Suite to better understand and fix redactions in text documents, where the first part of the pipeline detects redactions in PDF documents. Redactions are detected by identifying gaps between pairs of words that are larger than a single space character, and that consist of more non-white- than white pixels, to reduce the number of false positives. A downside of this approach is that the document should contain information on the location of characters, something that is often not present for scanned-in documents.

A technique that is more suited for usage with scanned documents is proposed by van Heusden et al [13], who describe a method based on a combination of Optical Character Recognition (OCR) and morphological operations to perform the detection. This approach reaches an F1 of .79 with an average IoU (the segmentation quality) of the correctly identified redactions of .86 (see Table 3). However, the fact that the model relies on handcrafted rules means that it is difficult to adapt the model to unseen scenarios, for example when new redaction types are introduced.

### Neural image segmentation

In the domain of Computer Vision, it is common to use pre-trained models such as VGG16[23] or ResNet[11] trained on large datasets of images, and to finetune the architectures with a domain-specific dataset. This is also the case for Document Object Detection (DOD), the task of locating and identifying various types of elements in documents, for example figures, tables, paragraph heading, etc. Naturally, redacted text detection is also a DOD task.

One such model that adapts a pre-trained model for DOD tasks is Figure Caption Extract Net (FCENet), developed by Liu et al [20]. The model architecture is based on the BlendMask architecture [6], which combines coarse object detections with fine-grained predictions based on attention. FCENet contains the addition of horizontal and vertical attention in the fine-grained detection step and the addition of separate prediction towers for figure and caption detection to adapt it to the task of document-image segmentation. The FCENet model is compared to BlendMask, Yolact and Mask R-CNN, three other neural instance segmentation models [5, 6, 12], and it outperforms all three methods in terms of both Average Precision and F1.

Another example of a general-purpose CNN model adapted for the task of document segmentation is the model proposed by [2]. The model is an adaptation of a Mask R-CNN model, adding a segmentation module to perform both object detection as well as instance segmentation. The method is compared to Faster R-CNN and Mask R-CNN models on the Historical Japanese Dataset (HJD) and PublayNet datasets. The Mask R-CNN model that is adapted for document-image segmentation outperforms both the Faster R-CNN and pre-trained Mask R-CNN models on the detection and segmentation of the majority of the selected categories, in terms of mean average precision.

The *DocSegTr* model from Biswas et al [3] is a further improvement of this Mask R-CNN model, where part of the architecture is replaced with a Transformer model. The DocSegTr model is able to better handle the segmentation of larger image elements such as table and figures, but has slightly worse performance for small image elements.

Huang et al [15] propose *LayoutLMV3* for various text-centric and image-centric tasks including document image classification and document layout analysis. The model is also based on the Transformer architecture, but differs from previous approaches by including textual information obtained from OCR. Both modalities are encoded using Transformers, and combined by using a multimodal Transformer. The addition of textual input means the model is better suited for tasks that require text understanding, such as the classification of individual items on receipts. The model was compared to various other models including UDoc and $\text {DiT}_{\text {BASE}}$ [17], and outperformed these methods on the majority of the image analysis datasets used in the research.

To conclude, the task of Document Object Detection has seen a multitude of well performing models, based on the Mask R-CNN and Transformer architectures, where most models differ in what type of architecture is used in which part of the model. As both types have different behaviour based on the specific dataset used, we use both a Mask R-CNN model as well as Mask2Former (a Transformer based model) in this research.

## Methodology

In this section we will discuss the dataset created for this research, the annotation process, and provide brief descriptions of both the Mask R-CNN and Mask2Former models. We also briefly describe a variant of the *Edact-Ray* model, adapted for usage with scanned-in documents.

### Data

**Fig. 1:**
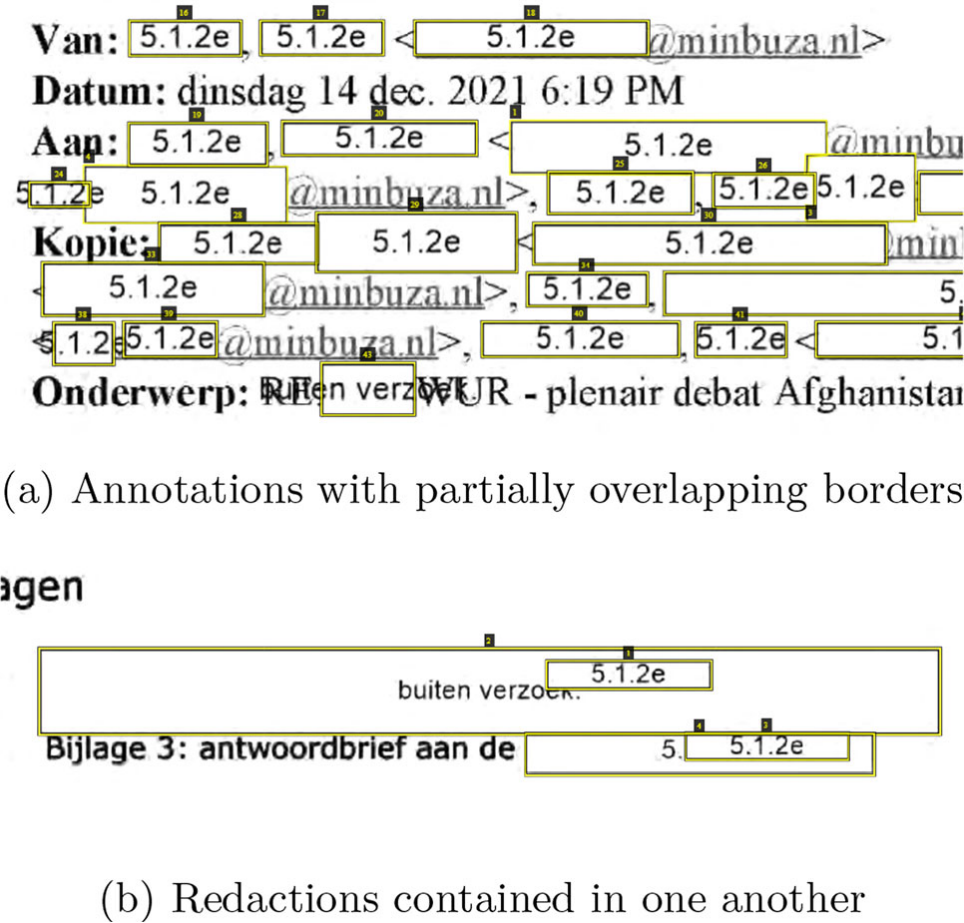
Examples of the annotations of borders in the VGG Image Annotator tool. Note how yellow annotation lines tightly follow the redaction borders instead of going through any partially overlapping redactions

**Fig. 2:**
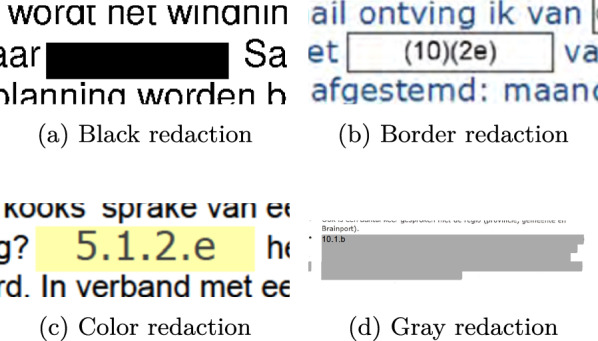
Examples of the different types of redaction in the dataset. The codes in the redactions are not type dependent. The color redaction can appear in different colors. The gray redactions can appear in different shades of gray

**Fig. 3:**
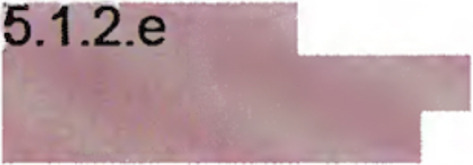
Redaction of a signature

To train and evaluate the developed models we use a dataset consisting of released FOIA requests from the Dutch government. All documents are in the Dutch language. The statistics of the dataset are presented in Table 1, both in terms of the number of pages and the number of annotated redactions. The pages without annotations have been added to mimic a realistic scenario in which such pages frequently occur. The redactions in the dataset have been classified into four possible types, namely *Black*, *Border*, *Color* and *Gray*, with examples of each of them shown in Figure 2. Although not explicitly classified as separate types of annotation, the dataset also contains ‘difficult’ redactions where the lines of redactions are quite faint, or annotations are of a particular unusual shape, as can be seen in Figure 3, where a signature has been redacted. The dataset was annotated using the VGG Image Annotator tool [9] by three annotators. The redaction blocks with humanly visible gaps were annotated as separate redactions and touching blocks on separate lines were annotated as a single redaction. The lines of overlapping redaction blocks are tightly followed so that there is no overlap in annotations for those blocks, see Figure 1a. In cases where boxes overlap such as in Figure 1b, only the redaction that contained the other redactions was annotated. The annotation was thus done in such a manner that ground truth segments are never overlapping, but may touch each other. For the experiments a dataset split of $70\%/30\%$ for the train- and test set was used.

**Table 1:** The number of pages and annotated redactions per redaction type in our annotated dataset

Redaction Type	Number of pages	Number of redactions
Black	314	3,914
Border	170	2,535
Color	83	1,660
Gray	381	3,242
No redaction	516	0
**Total**	**1,464**	**11,351**

### Models

#### Mask R-CNN model

The Mask R-CNN model is an image segmentation model based on convolutional neural networks, developed by He et al [12], as an extension to the Faster R-CNN model [10].

The Mask R-CNN model consists of two stages; the first stage is the Region Proposal Network (RPN) which proposes regions of interest (RoI) from input images and the second stage extracts features from these RoIs and performs classification and bounding-box regression on them. In parallel to the second stage, the Mask R-CNN model also outputs binary segmentation masks for each RoI, allowing for both semantic- and instance segmentation. The Mask R-CNN model can be instantiated with multiple architectures; a convolutional backbone architecture for the feature extraction (over an entire image) and a network head for the bounding-box recognition and mask prediction of each RoI. For the implementation of the model we used the Detectron2 library [25] from Meta with the ResNeXt-101-32x8d [26] and Feature Pyramid Network (FPN) [19] backbones for the feature proposal and mask predictions steps respectively, following Biswas et al [2]. We use a model trained on the ImageNet dataset [18], as this model yielded the best performance^4^. The model was trained with a learning rate of 0.001 without decrease, the default 1,000 warm-up iterations and a maximum of 5,000 optimization steps (roughly 15 epochs). We did not use random flip nor did we filter out images without annotations from the training data.

#### Mask2Former

The Mask2Former model was introduced by Cheng et al [8], and is an extension of the MaskFormer model [7]. The model consists of three main components, namely a backbone that extracts low-level features from input images, a pixel decoder that enhances these low-level feature maps, and a Transformer decoder module that outputs binary masks. The model can perform universal segmentation, meaning it can simultaneously perform semantic- and instance segmentation, also referred to as Panoptic segmentation. This architecture bears a resemblance to the DocSegTr model proposed by Biswas et al [3], however the generation of the feature maps is now solely performed by a Transformer, and the generation of the final output masks combines the pixel decoder and Transformer decoder, instead of using features from the backbone. For the implementation of the Mask2Former model, we used the Mask2Former library from Meta[7] and a model pre-trained on instance segmentation for the MS COCO dataset, as this proved more successful than finetuning the pixel decoder and Transformer decoder on our dataset. We used a Swin-T model backbone[21], 5,000 optimization steps (roughly 15 epochs), a learning rate of 0.0001, and a batch size of 2, and performed training an a single GPU.

#### OCR & morphology baseline

van Heusden et al [13] introduce a baseline that combines OCR with morphological operations to remove all the text from an image, and subsequently performs contour detection (with some filtering on object sizes and shapes) to extract redactions from scanned-in documents. The image is first preprocessed using erosion and dilation techniques, after which OCR is used to obtain the locations of characters in the image, which are then removed. Finally, contour detection is performed to extract the remaining shapes, which are filtered based on size and orientation. We use the same hyperparameters as the original paper for our dataset as this yielded the best performance.

#### Edact-Ray on scans

As previously mentioned, The Edact-Ray tool from Bland et al [4] uses a detection method based on the location of characters and "too long" spaces in a document to detect inline redactions. This simple baseline using an appealing heuristic worked well on the digital court proceedings used in the original paper, and thus seemed a good candidate for a strong baseline. However, our redactions are in scanned documents, and so we had to adapt their method. We discuss our adaptations and argue why the heuristic does not work well on scans. For this reason, we did not include results for this model in the paper. It is not guaranteed that character location information is present in scanned-in documents, and thus OCR has to be performed to obtain it. Moreover, some documents can contain redactions spanning multiple lines, which cannot be detected by simply using horizontal gaps between words. In an attempt to adapt the Edact-Ray detection method for scanned documents, we propose a version of the algorithm that consists of the following steps.

1. Detection of word location using OCR, using a confidence threshold of 0.65 to mitigate false positive text.
2. Detection of inline redaction by comparing word boundaries, and marking gaps that are larger than twice the size of an average space.
3. Detection of multiline redaction by comparing the differences in height between consecutive sentences, marking gaps that are larger than twice the average character height.
4. Filtering of redactions by requiring at least half of the pixels to be non-white, to avoid false positives (for example newlines between paragraphs or text indentation).

Although the proposed approach works somewhat for the black- and color redactions (with a segmentation quality of 0.62 and 0.58 and a recognition quality (F1) of 0.16 and 0.30 for black- and color redactions respectively), the model is not able to detect border redactions, largely because of two reasons. First, the border redactions often contain codes, and as such there will not be a large gap between consecutive words (see Figure 1a). Although these codes could be filtered, the other problem is that these redaction boxes contain predominantly white pixels and thus will not pass through the color constraint. Lifting this constraint however is not an option, as it would lead to a large amount of false positives.

**Fig. 4:**
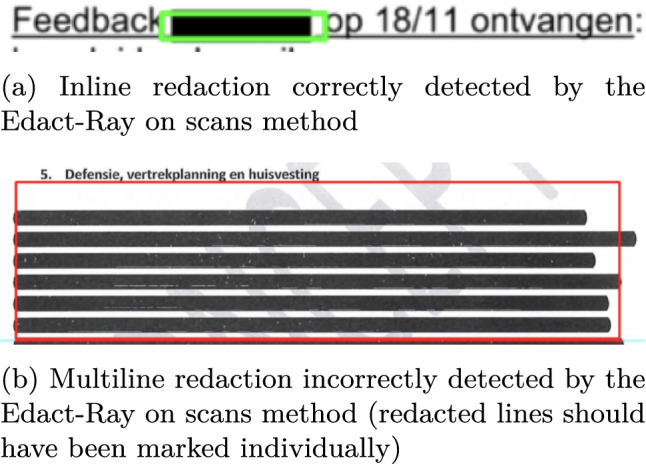
Examples of inline- and multiline redactions identified by the Edact-Ray on scans method

Another shortcoming of the method can be seen in Figure 4b. Although the model apparently correctly identified a multiline redaction, the redacted lines should have been annotated as separate redactions, not as a single block. However, since none of these lines contain any text, the model is incapable of making this distinction. This is also reflected in the scores, as the recall of the model is lower than the precision for all classes The aforementioned problems all stem from the fact that, in its core, the algorithm relies on a sufficient amount of textual information being present to detect redactions. If however a significant amount of text has been redacted, this information is not available, and thus the model will not be able to make accurate detections, even with more sophisticated rules.

### Mask post-processing

Both the Mask R-CNN and Mask2Former models output masks that can possibly overlap, which is not allowed by the assumptions underlying evaluation with the Panoptic Quality methodology. To remove these overlapping predictions, we follow the procedure described by Kirillov et al [16], where predicted masks are sorted by their confidence and predictions with a confidence lower than a set threshold are removed. If two masks overlap more than a set threshold, the least confident mask is discarded, otherwise the overlapping portion is removed from both masks, and both masks are kept. We used a threshold of 0.5 for both the confidence- and overlap thresholds.

### Computational resources

Both the Mask R-CNN and the Mask2Former models were trained using one Nvidia Tesla P40 GPU with 24 GB DDR5 memory. The total training time for both models was roughly one and a half hours for the complete dataset, including pages without redactions.

### Evaluation

Traditionally, Average Precision (AP) has been used to measure and compare the performance of instance segmentation models. The average precision is based on ground truth and predicted *objects* and is calculated by sorting predicted objects on their confidence score, and calculating precision and recall by starting at the most confident prediction, and including more and more samples with lower confidence. True Positives, False Positives and False Negatives are defined by using Intersection-over-Union (IoU) thresholded at 0.5, and average precision is then defined as the area under the precision-recall curve. Different benchmarks use slightly different definitions, by for example also taking averages over a set of IoU thresholds [18]. Regardless of the specific method, a major downside of this evaluation paradigm is that it does not measure the quality of the prediction, i.e. how close the prediction is to the ground truth. To remedy this issue, Kirillov et al [16] have proposed the panoptic quality (PQ) metric, which, like AP, operates on ground truth and predicted objects.

The PQ metric uses the IoU to calculate matches between predicted and ground truth objects, and defines True Positives (TP), False Positives (FP) and False Negatives (FN) over sets of objects. Given sets of ground truth and predicted objects T and H respectively, the classes TP, FP and FN can be defined as follows:

$$\begin{aligned} TP&= \{ (h, t) \in H \times T \mid IoU(h, t)>0.5 \} \\ FP&= H \setminus dom(TP) \\ FN&= T\setminus range(TP). \end{aligned}$$

Precision, recall and F1 are then defined as usual. The F1 score is referred to in [16] as the Recognition Quality (RQ). The segmentation quality (SQ) indicates how precisely the truely predicted segments match the ground truth. It is defined as the average IoU of the set of TPs. We will report precision, recall, F1 and the segmentation quality score.

### Code availability

All the code and the data used in this research are publicly available on GitHub^5^.

## Results

This section contains our main results: the performance of the neural models and the baseline on the "regular" train and test set (with at least one redaction on every page) (Section 4.1) and on the extension of that set with hard negatives (pages without redactions) (Section 4.2). In Section 4.3 we look at the effect of reducing the number of training samples for the Mask R-CNN and Mask2Former models.

### Redacted text detection

**Table 2:** SQ, Precision, Recall and F1 scores of the OCR+Morphology, Mask R-CNN and Mask2Former models on pages that contain redactions, reported per redaction type, where bold indicates the redaction type with the best score for the specific model and metric. The support is the number of redactions for each redaction type in the test set

	OCR+Morphology				Mask R-CNN				Mask2Former				
	SQ	P	R	F1	SQ	P	R	F1	SQ	P	R	F1	Support
Black	.85	**.93**	**.90**	**.92**	.85	**.97**	**.98**	**.98**	.84	.95	**.95**	**.96**	1,338
Color	.87	.84	.87	.85	**.86**	.97	.95	.96	.85	**.97**	.90	.93	542
Gray	.83	.73	.65	.69	.85	.91	.95	.93	**.85**	.94	.93	.93	795
Border	**.88**	.83	.44	.57	.86	.97	.88	.95	.83	.93	.82	.88	911

Table 3 reports on our main experiment: comparing the three models on the test set consisting of pages with at least one redaction. We report the segmentation quality (SQ), precision, recall and F1 scores for the rule-based OCR+Morphology, Mask R-CNN and Mask2Former models. Both neural models outperform the rule-based OCR+Morphology model on precision, recall and F1. Mask R-CNN is the best performing system concerning detection, but the OCR+Morphology model is best in precisely segmenting the redactions, witnessed by the highest SQ score.

We now zoom in and report the performance of the models for the different types of redaction exemplified in Figure 2. Table 2 shows the performance of the models per redaction type. Bold indicates the redaction type with the best score for the specific model and metric. The more traditional redaction styles, black and color, are the easiest to detect for all models. The baseline has much lower performance, in particular recall, on the gray and border types. The neural models, especially Mask R-CNN, have a more constant high performance on all four types. for the recall on the border class which is remarkably lower for the OCR+Morphology and Mask2Former models.

**Fig. 5:**
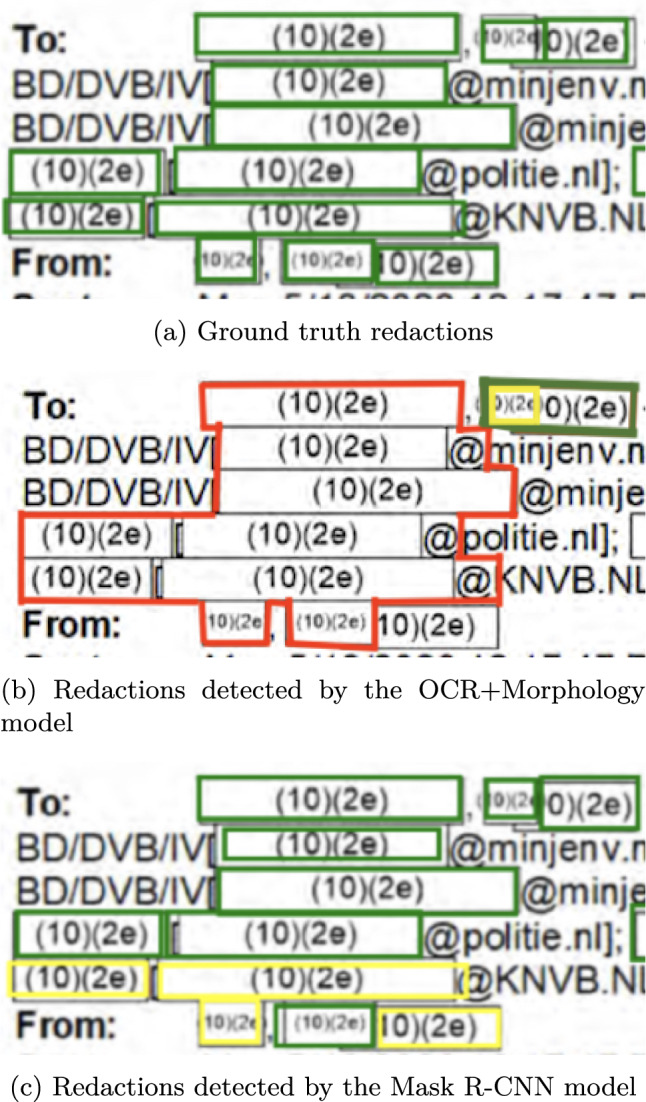
An example of border redactions being incorrectly fused by the OCR+Morphology model and correctly separated by the Mask R-CNN model. (In all figures, green indicates correct predictions, red indicates false predictions, and yellow indicates missed predictions)

For the border redactions, the main reason for redactions being missed was the model incorrectly fusing multiple redactions into one, as shown in Figures 5a and 5b. This type of redaction, where the redactions are very close together, occurs often for the border class, explaining the low recall of the models on this class. The Mask R-CNN and Mask2Former models handle this type of redaction much better (see Figure 5c), however some border redactions are still missed.

The Mask R-CNN and Mask2Former perform more or less similar on all redaction types except for the recall on the border class whichthat the recall on the border class is .06 lower for Mask2Former. No clear cause for this difference could be found, apart from the fact that the Mask R-CNN model picked up more redactions in situations where a lot of redactions were close together. Figure 7 contains a challenging example where border redactions have to be recognized inside a table (with a border as well).

**Fig. 6:**
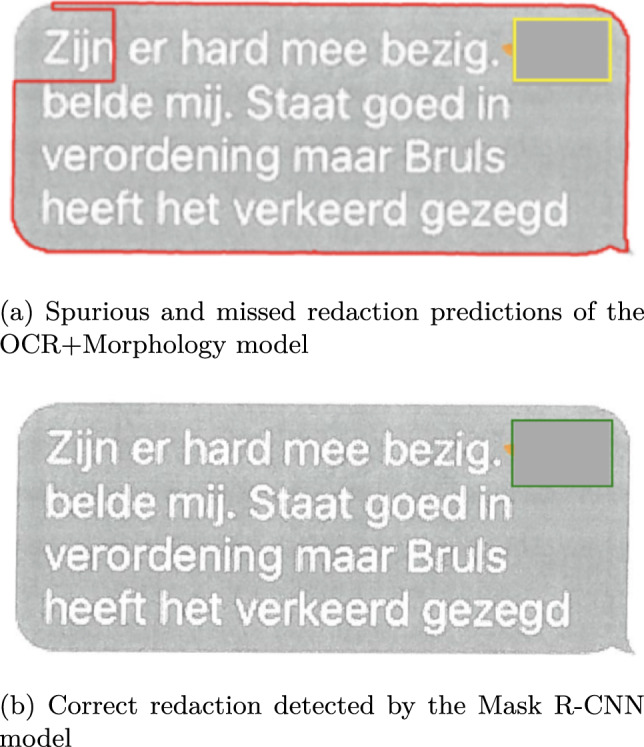
An example of a gray redaction being missed by the OCR+Morphology model, and correctly separated by the Mask R-CNN model

We did not find a singular cause for the poor performance of the OCR+Morphology model on the gray redactions. There are several cases of redactions inside tables that are not detected (false negatives), but also instances in which for example gray scans of Whatsapp messages were mistaken for redactions (see Figure 6a).

**Fig. 7:**
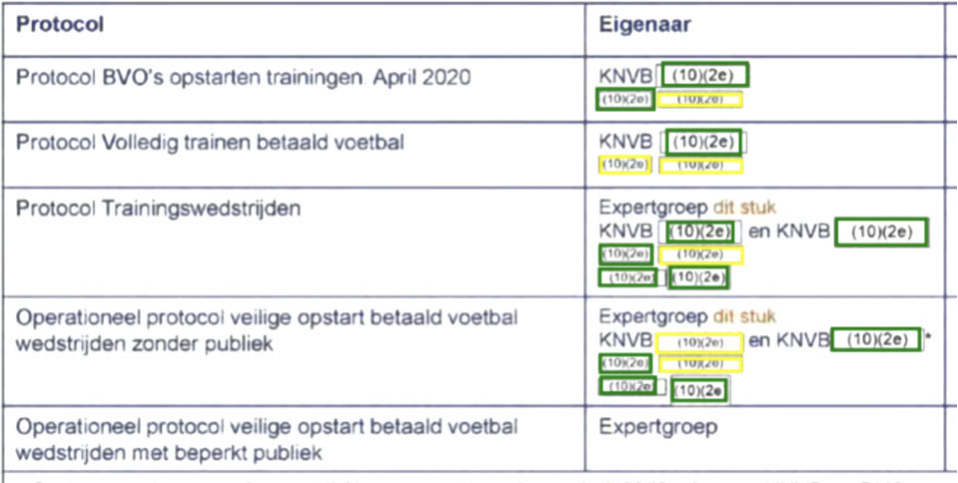
Predictions of the Mask R-CNN model on a table that contains border redactions

All three models perform comparably in correctly segmenting the true positives, with SQ values between .83 and .88. (Note that SQ is always between .5 and 1 by definition of a true positive.) This is partly due to the ground truth annotations, as some of the redactions are very small, and it is difficult to place a bounding box exactly on the border of the redaction. Moreover, in the case of small objects, a discrepancy between two masks can have a large effect on the SQ metric.

### Adding pages without redactions

For this experiment, we added pages without annotations to the dataset, and evaluate the differences in model performance compared to the dataset in which every page contains at least one redaction. Of course this better resembles real-world data, which often contains pages with standard boilerplate content. We expect that the addition of redaction-free pages leads to lower recognition scores, in particular we expect more false positives. First, we added these pages without annotations to the test set only, to investigate to what extent the models were able to handle these pages without being explicitly trained with redaction-free pages. For the Mask R-CNN and Mask2Former models, this led to a significant amount of false positive detections on these empty pages (84 and 97 respectively), given that the test set contains 155 pages without redactions. As the OCR+Morphology model contains no training step and thus there is no difference with the previous experiment, it was not included in this experiment.

We now look what happens when we also train the neural models with pages without any redaction. Table 4 is like Table 3 except that now the models are trained and tested on the extension of the earlier used train and test sets with pages without redactions. Overall performance of all three models decreases, with the decrease being largest for the OCR+Morphology method. Only the precision of the OCR+Morphology model is affected, as no additional training was performed, so the predictions on the pages with redactions remained unchanged.

**Table 3:** Segmentation Quality, Precision, Recall and F1 of the OCR+Morphology, Mask R-CNN and Mask2Former models on pages that contain redactions (N_pages=284, N_redactions=3,586)

Model	SQ	P	R	F1
**OCR+Morphology**	**.86**	.85	.72	.78
**Mask R-CNN**	.85	**.96**	**.94**	**.95**
**Mask2Former**	.84	.95	.90	.93

**Table 4:** SQ, precision, recall and F1 scores for the rule-based, Mask R-CNN and Mask2former models on the test set that contains pages without redactions (N_pages=439 (155 without redactions), N_redactions=3,586)

	SQ	P	R	F1	Empty page FPs
Rule-based	**.86**	.62	.70	.66	1,127
Mask R-CNN	.84	.90	**.92**	**.91**	52
Mask2Former	.84	**.94**	.83	.89	**2**

**Fig. 8:**
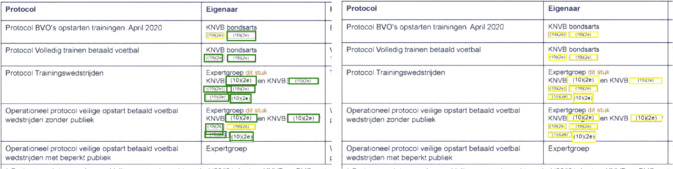
Comparison of the versions of the Mask2Former model trained only on pages with redactions (left) and on pages with and without redactions (right)

**Fig. 9:**
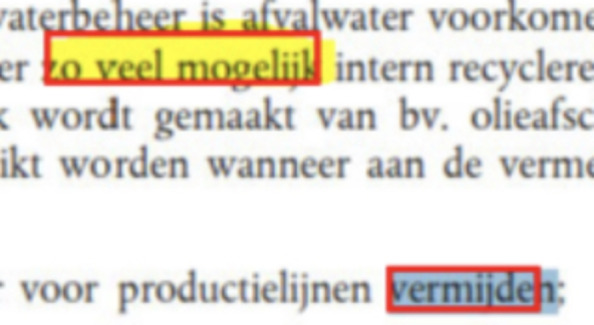
Spurious detection of highlighted text by the Mask R-CNN model (after training on the complete train set)

The Mask2Former model has almost no false positives on the pages without redactions, while Mask R-CNN has 52. Many of these false positives were cases where there was text inside a table, or when there was highlighted text, such as in Figure 9.

The number of extra false positives in the pages without redactions in the last column shows that the two neural models are more robust to realistic input with pages without redactions. The large number of false positives for the OCR+Morphology model is due to the fact that, through the text filtering and contour detection steps, it will pick up rectangular elements in pages, such as footers, parts of illustrations, and individual cells in tables.

The low amount of false positives does come with a trade-off for the Mask2Former model however, as the recall is reduced from .90 to .83. The largest decrease in recall was observed in the border class, an example of which is shown in Figure 8. Here we see a dramatic decrease in recall when the model is also trained on pages without redactions. The likely explanation for this is that the model has learned to ignore tables in pages (if they do not contain redactions themselves), and that this has caused the model to not detect some redactions that might be similar to tables (large border redactions with text).

### Influence of the number of training samples

We now investigate the influence of the number of training samples on the performance of the two neural models. We take stratified subsets of the complete dataset (including pages without annotations) of $10\%$, $20\%$, $40\%$, $60\%$, $80\%$ and $100\%$ percent of the number of pages. We train both the Mask R-CNN and Mask2Former models on those subsets of the dataset, and evaluate on the complete test set.

**Fig. 10:**
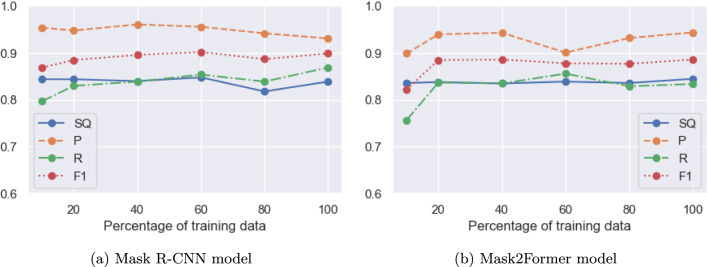
Performance of the Mask R-CNN and Mask2Former models in terms of SQ, P, R and F1 for $10\%$, $20\%$, $40\%$, $60\%$, $80\%$ and $100\%$ of the training data (in terms of number of pages)

Figures 10a and 10b show the progression of performance for both neural methods when the amount of training data is increased. Interestingly, the performance of both models is relatively consistent across different sizes, and even with only 20 percent of the original training set (roughly 12,200 annotations), both models achieve close to their final performance. This shows that for this specific task, the pre-training on MS COCO is very effective in training the models for instance segmentation, and that only very little data is needed to adapt these models to the specific task of redaction detection.

### Post-processing model output

Although both the Mask R-CNN and the Mask2Former models show a significant improvement over the rule-based model when applied to pages that contain no redactions, they still make some mistakes. Some (like the one shown in Figure 9), could be relatively easily removed by performing OCR on the model output, and filtering the predictions based on this. Since the Mask2Former model only had two false positives, we will focus on the Mask R-CNN model for this experiment. Of the 52 false positives yielded by the Mask R-CNN model, 22 of these false positives contained readable text according to Tesseract (with a confidence threshold of .70), and could thus benefit from a post-processing approach. Because some of the legitimate redactions in the dataset contain text (such as a code, or the reason for redaction) we cannot simply filter out all redactions that contain text, but rather have to use regular expressions and (fuzzy) string matching to filter out often-used codes and phrases. Using this approach, 21 of the 22 false positives detected by the Mask R-CNN model could be filtered out. However, this does come at the cost of having more false negatives, with 585 false negatives for the Mask R-CNN model after filtering, compared to 468 false positives in the original setting. A substantial portion of these false negatives came from redactions where Tesseract was not able to extract text due to the small size of the redaction (such as the bottom-left redaction in Figure 5cs.) Although the precision of the model is increased from .90 to .96, the recall of the model has dropped from .92 to .84 resulting in a decrease of the F1 score of the model from .91 to .89. Although the drop in performance is relatively small, and in cases where precision is important this approach might be reasonable, it introduces another layer of complexity in the detection pipeline, where specific rules have to be crafted to strike a balance between false positives and false negatives.

## Discussion & future work

Although both neural methods outperform the OCR+Morphology model in both experiments, they only operate on the images of pages, and do not explicitly use the textual information. We tried incorporating textual information using Tesseract as a post-processing step to improve model performance, but found that this approach resulted in a net decrease in model performance.

Future work in this direction could look into incorporating textual information in a more sophisticated manner, for example by using a multi-modal approach where a textual- and visual model are trained simultaneously to recognize which when text in a redaction is acceptable (being a code for example), or when this indicates a false positive.

## Conclusion

We compared two neural instance segmentation models with a strong rule-based baseline for the detection of redacted pieces of text in documents. Models were tested and trained on documents released after a request based on the Freedom of Information Act. Both neural methods significantly outperform the rule-based method: they pick up more redactions, make fewer mistakes and are also more robust to realistic data containing pages without redactions. The Mask R-CNN model performed best, with a precision and recall of .96 and .94, respectively when trained and tested on pages with redactions. This dropped slightly to .90 and .92 when adding hard negatives in the form of pages without redactions. We additionally conducted an experiment to filter the output of the Mask R-CNN model using Tesseract to reduce the number of false positives. We found that although effective in reducing false positives, this approach also increased the number of false negatives, resulting in a net drop in performance of the resulting model. We ported the simple and appealing rule-based baseline from Bland et al [4] which worked well on digital documents to scanned documents but found that the used heuristic is too brittle for this more "dirty data", leading to subpar performance compared to the other methods.

## Footnotes

1 https://www.nj.gov/grc/custodians/redacting/.

2 https://www.justice.gov/oip/blog/foia-post-2008-oip-guidance-segregating-and-marking-documents-release-accordance-open.

3 https://www.w3.org/TR/wcag-3.0/.

4 https://github.com/facebookresearch/detectron2/blob/main/MODEL_ZOO.md.

5 https://github.com/RubenvanHeusden/NeuralRedactedTextDetection.
